# Ultrasensitive dopamine detection with graphene aptasensor multitransistor arrays

**DOI:** 10.1186/s12951-022-01695-0

**Published:** 2022-11-24

**Authors:** Mafalda Abrantes, Diana Rodrigues, Telma Domingues, Siva S. Nemala, Patricia Monteiro, Jérôme Borme, Pedro Alpuim, Luis Jacinto

**Affiliations:** 1 International Iberian Nanotechnology Laboratory, 4715-330 Braga, Portugal; 2 Life and Health Sciences Research Institute (ICVS), School of Medicine, University of Minho, 4710-057 Braga, Portugal; 3 ICVS/3B’s—PT Government Associate Laboratory, 4710-057 Braga/Guimarães, Portugal; 4 Physics Center of Minho and Porto Universities (CF-UM-UP), 4710-057 Braga, Portugal; 5 Present Address: Faculty of Medicine of the University of Porto (FMUP), 4200-319 Porto, Portugal

**Keywords:** Graphene, Field-effect transistor, Aptasensor, Dopamine, LOD, Parkinson’s disease

## Abstract

Detecting physiological levels of neurotransmitters in biological samples can advance our understanding of brain disorders and lead to improved diagnostics and therapeutics. However, neurotransmitter sensors for real-world applications must reliably detect low concentrations of target analytes from small volume working samples. Herein, a platform for robust and ultrasensitive detection of dopamine, an essential neurotransmitter that underlies several brain disorders, based on graphene multitransistor arrays (gMTAs) functionalized with a selective DNA aptamer is presented. High-yield scalable methodologies optimized at the wafer level were employed to integrate multiple graphene transistors on small-size chips (4.5 × 4.5 mm). The multiple sensor array configuration permits independent and simultaneous replicate measurements of the same sample that produce robust average data, reducing sources of measurement variability. This procedure allowed sensitive and reproducible dopamine detection in ultra-low concentrations from small volume samples across physiological buffers and high ionic strength complex biological samples. The obtained limit-of-detection was 1 aM (10^–18^) with dynamic detection ranges spanning 10 orders of magnitude up to 100 µM (10^–8^), and a 22 mV/decade peak sensitivity in artificial cerebral spinal fluid. Dopamine detection in dopamine-depleted brain homogenates spiked with dopamine was also possible with a LOD of 1 aM, overcoming sensitivity losses typically observed in ion-sensitive sensors in complex biological samples. Furthermore, we show that our gMTAs platform can detect minimal changes in dopamine concentrations in small working volume samples (2 µL) of cerebral spinal fluid samples obtained from a mouse model of Parkinson’s Disease. The platform presented in this work can lead the way to graphene-based neurotransmitter sensors suitable for real-world academic and pre-clinical pharmaceutical research as well as clinical diagnosis.

**Supplementary Information:**

The online version contains supplementary material available at 10.1186/s12951-022-01695-0.

## Introduction

Neurotransmitters are molecules indispensable for the communication between neurons and play a critical role in brain function. The accurate detection of neurotransmitter concentrations in the brain and biological samples is of great importance for neurobiology research and developing novel diagnostic and therapeutic approaches for brain disorders affecting neurotransmitter levels and dynamics. A significant neurotransmitter is dopamine (3,4-dihydroxyphenethylamine), which has essential roles in the human brain and body, regulating several physiological processes involved in motor function, memory, motivation, arousal, and reward [1]. Abnormal alterations in the levels of dopamine can have severe consequences and underly brain disorders such as Parkinson's Disease (PD), Alzheimer's Disease (AD), Schizophrenia, Attention Deficit and Hyperactive Disorder (ADHD), and substance addiction [1–3]. These mental disorders are associated with a high personal, societal and economic burden [4–7] and have been growing worldwide in terms of prevalence and related disability. For example, PD and AD, the two most common neurogenerative disorders, have no cure or preventive neuroprotective therapies, and their current estimated worldwide prevalence of 1–2% is expected to grow within the next decades [5, 7]. Thus, the ability to detect physiologically relevant dopamine concentrations by high-throughput approaches in the brain or brain-derived biological samples can accelerate the development of early diagnostics and improved neurotherapeutics for these disorders. However, conventional analytical methodologies to monitor and detect dopamine, which include enzyme-linked immunosorbent assays (ELISA), high-performance liquid chromatography (HPLC), capillary electrophoresis, and spectroscopy, rely on large-scale, expensive equipment or require laborious sample preparation and long detection cycles [8, 9]. Novel emerging approaches have focused on miniaturized biosensors, primarily based on catalytic and electrochemical reactions, but have mostly lacked relevant selectivity and sensitivity [10, 11]. Current reported limits of detection (LOD) for dopamine sit above the femtomolar range [12, 13], with most sensors presenting narrow working ranges between nM and µM concentrations [10, 11], which limits their ability to reliably detect physiological dopamine concentrations from small volume samples. Additionally, many novel biosensors for dopamine and other neurotransmitters detection have complicated fabrication processes [10, 11] that pose constraints on miniaturization and integration, thus limiting dissemination, reproducibility, and the development of relevant research tools and point-of-care devices.

Graphene-based biosensors have been attracting growing attention due to their extremely high sensitivity based on graphene’s unique electronic properties, high chemical and mechanical stability, and biocompatibility [14–17]. Graphene field-effect transistors (gFETs), in particular, take advantage of graphene’s exceptionally high carrier mobility and surface-to-volume ratio to permit high signal-to-noise transduction of biodetection events through electrostatic gating [17–19]. Because the gFETs' transduction depends on the field-effect modulation based on different local doping mechanisms, charge carrier scattering, and dielectric environment [20–22], they can be designed and tuned according to application demands. The graphene channel on gFETs can also be functionalized through surface chemistry with biorecognition elements such as enzymes, antibodies, DNA, and aptamers for selective biodetection [14, 23–25]. Since target detection events lead to electrostatic gating of the channel by the biorecognition element or by the target, graphene's high surface-to-volume ratio is especially advantageous for enhanced sensor sensitivity [17–19].

Consequently, a wide range of selective biosensing applications with gFETs, including protein and DNA detection in optimized buffers and samples prepared from body fluids such as blood, sweat, or saliva, has been reported in the last decade [15, 26, 27], including by us [26, 28]. However, despite promising operation in controlled conditions, gFET biosensors, like any ion-sensitive FET, suffer dramatic sensitivity reductions in biological conditions or media. The decreased sensitivity occurs due to the reduction of the Debye length [29], i.e., the distance over which the local electric field can modulate charge carriers in the graphene channel due to ionic strength increase. Approaches to overcome these limitations in FET-based biosensors have included electronic tunning by adding a floating gate configuration [30], removal of the surface excess ion population [31], channel morphology alterations [32, 33], and reduction of the biorecognition element size [32], with the latter being a promising approach for gFET biosensors to preserve crystalline graphene's unique properties without considerably increasing fabrication complexity.

Additionally, many of the recently proposed graphene biosensors do not address issues relating to fabrication methods' replicability and scalability and the desired measurements' stability and reproducibility. Thus, despite significant efforts, the deployment of graphene-based point-of-care devices and reliable academic and pharmaceutical research tools has been limited. Wafer-scale graphene synthesis and fabrication of graphene biosensors with replicable methodologies can improve device uniformity and facilitate integrated designs with multiplexed parallel assays for high-throughput measurements [14, 34–37], a desired feature for real-world neurotransmitter sensors.

This work proposes a biosensing platform that permits ultrasensitive and selective dopamine detection, yielding stable and reliable results by high-throughput single-sample measurement replication. Micron-sized electrolyte-gated field-effect graphene transistors (EG–gFETs) were fabricated with reproducible methodologies optimized at the wafer level and integrated into miniaturized multitransistor array (gMTA) chips. These arrays were converted into aptasensors by functionalizing the EG–gFETs with a short-strand dopamine-specific DNA aptamer. The array format introduced here allows single-sample measurement replication within each gMTA, producing robust average data and reducing sources of measurement variability. By taking advantage of this novel feature, the high mobility of graphene's charge carriers, and the effective screening of the aptamer's charge redistribution upon dopamine binding, a record LOD for dopamine and wide sensing ranges are reported. The obtained LOD of 1aM is three orders of magnitude lower than the lowest dopamine LOD previously reported. Furthermore, we show that these gMTAs can detect minimal changes in dopamine concentration in small working volume (2 µL) biological cerebrospinal fluid (CSF) and brain homogenate samples from an animal model of PD. This feature is highly pertinent for developing novel point-of-care devices and research tools that require stable high-throughput detection of physiologically and clinically relevant dopamine concentrations.

## Results and discussion

### Fabrication of graphene multi-transistor arrays (gMTA)

**Fig. 1:**
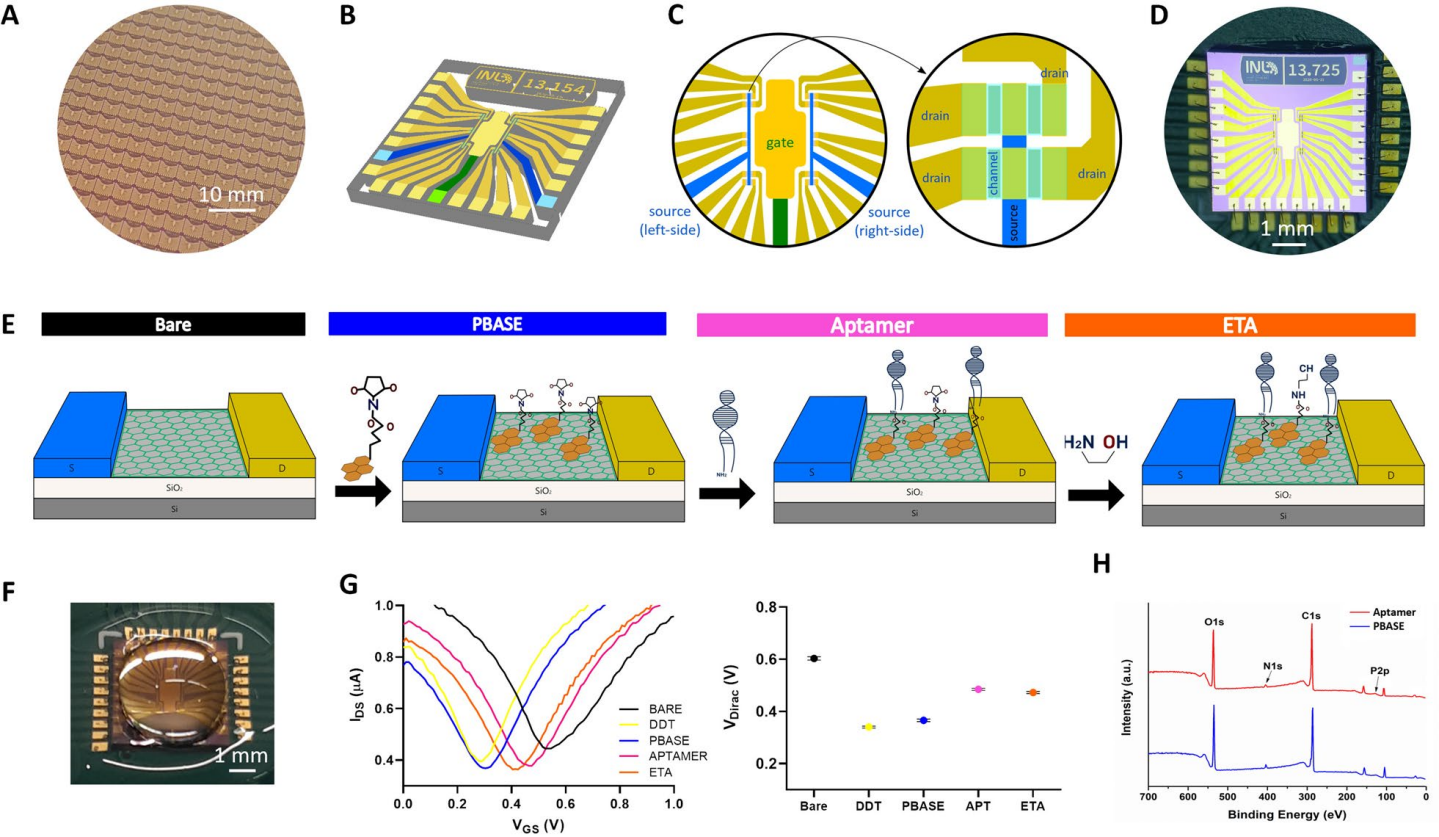
Graphene aptasensor multitransistor arrays (gMTAs) for dopamine detection. **A** Detail of wafer containing 784 fabricated gMTAs. **B** Schematic illustration of one gMTA chip with 20 electrolyte-gated graphene field-effect transistors (EG–gFETs) and respective interconnect lines and pads. **C** Schematic illustration of a gMTA's sensor area with EG–gFETs sharing a co-planar integrated gate electrode (golden central region and green interconnect line), individual drain electrodes (yellow contacts and interconnect lines), and 2 common source electrodes for every 10 groups of transistors (blue contacts and interconnect lines) (left). Detail of 4 transistors with 4 graphene channels (light blue) connecting 4 independent drain electrodes (yellow) and a common source electrode (dark blue) (right). **D** Photograph of one gMTA wire-bonded to a custom-made PCB for electronics interfacing. **E** Schematic of graphene biofunctionalization process for each EG–gFET in the gMTAs: the exposed bare graphene channel (left) was initially passivated with a pyrene-derived crosslinker (PBASE) (center-left), followed by the addition of a dopamine-specific DNA aptamer that binds to PBASE (center-right) and of ethanolamine (ETA) for blocking unreacted PBASE (right) (N.B. schematic not to scale). **F** Photograph of a gMTA with a phosphate-buffered saline (PBS) droplet on top of the sensor area. **G** Representative transfer curves from one EG–gFET as measured in 1 × PBS after each biofunctionalization step (left). Average value of the charge neutrality point (V_DIRAC_) after each functionalization step for 200 transistors (data is mean ± sem) (right). **H** X-ray Photoelectron Spectroscopy (XPS) survey for graphene samples with PBASE (blue) and PBASE + aptamer (red)

High-throughput ultrasensitive detection of dopamine in real-world biosensing applications requires the realization of a miniaturized biosensor that can yield stable measurements in a wide range of sensing media and contexts. Reproducible wafer-scale high-yield fabrication methods are also required to ensure the replicability of graphene-based biosensors. Therefore, an Si/SiO_2_ wafer containing 784 graphene multi-transistor arrays (gMTAs) with 15,680 electrolyte-gated graphene field-effect transistors (EG–gFETs), with a yield of approximately 80%, was fabricated for this work (Fig. 1A) (see details in Methods). Each 4.5 × 4.5 mm^2^ gMTA chip consists of an array of 20 EG–gFETs with individual gold drain electrodes connected to 2 common gold source electrodes, with groups of 10 transistors sharing a common source, and respective interconnect lines (Fig. 1B, C). This design leverages our previously published architecture, which includes an integrated receded electrolytic gold gate electrode [35, 38]. The integrated gate design facilitates the fabrication of compact devices for real-world applications compared with remote top gate designs, typically found in other FET-based biosensors that require an externally applied wired gate electrode to the electrolytic solution for modulation of the local electric field [39–42]. In our design, the large area in-plane gate also provides a uniform gating field for all transistors across the sample [26, 35]. Drain and source electrodes are connected by a 25 µm long and 81 µm wide single-layer graphene channel (Fig. 1C). Graphene was grown by chemical vapor deposition (CVD) (Additional file 1: Fig. S1), commonly used to produce large-area polycrystalline single-layer graphene sheets [43], and patterned by optical lithography. Dielectric passivation of source and drain electrodes was achieved with a 250 nm multi-stack passivation layer of SiO_2_/SiN_x_ that improves the impermeability to solvents during the following stages of transistors' functionalization and increases resistance to delamination in prolonged exposure to liquid solutions for biosensing applications [44]. Finally, the gMTA chips were mounted and wire-bonded to a custom-designed printed circuit board (PCB) for electronics interfacing (Fig. 1D). The overall fabrication method is highly reproducible at a low cost in a clean-room facility and optimized at the wafer level to preserve graphene's electronic properties, ensuring reproducible devices with high sensitivity. The integrated multitransistor array design allows simultaneous parallel measurements in different EG–gFETs in one single chip from a single sample. Although each EG–gFET in a gMTA chip provides independent measures of the analyte, averaging across transistors' replicates in a single measurement session produces robust averaged data, reducing possible sources of transistor-to-transistor and measurement variability (Additional file 1: Fig. S2). Additionally, because the EG–gFETs can be independently controlled, malfunctioning transistors can be electronically disconnected within a gMTA, not contributing to the final readout values. Thus, this parallel multi-sampling approach produces robust averaged data from single samples for stable and reliable detection regardless of sensing media and conditions.

### Biofunctionalization of graphene surface for gMTA aptasensor

The EG–gFETs’ graphene channel was functionalized with a dopamine-specific biorecognition probe to guarantee selective dopamine detection, and all remaining exposed surfaces in the device were passivated. A non-covalent biofunctionalization strategy was implemented for immobilizing a short-strand dopamine-specific DNA aptamer (Fig. 1E). This approach allows simultaneous sensitive transduction and effective screening within the Debye length. The gate electrode was initially passivated with a thin self-assembled monolayer (SAM) of dodecanethiol (DDT) to prevent adsorption onto the gold surface of any solution molecules. Functionalization of the graphene surface was then achieved with 1-Pyrenebutyric acid N-hydroxysuccinimide (PBASE) that non-covalently binds to graphene through *π*–*π* stacking of its aromatic side chains [45] (Fig. 1E). The use of non-covalent immobilization preserves graphene’s hyper-conjugated aromatic structure and exceptional electronic mobility [25], which is crucial for developing ultrasensitive biosensors. The succinimidyl ester group of PBASE then remains available to form a covalent bond with an amine-terminated biorecognition probe via nucleophilic substitution [20]. This approach was previously used to immobilize DNA probes in graphene-based biosensors successfully [26, 46]. For selective detection of dopamine, a DNA aptamer previously shown to have a high affinity for dopamine with a binding constant of 0.25 µM [46, 47] was immobilized in the EG–gFETs by binding to PBASE’s ester group via a 5′ extremity amine-terminated modifier (Fig. 1E). Finally, ethanolamine (ETA) was used to block any remaining unreacted PBASE after aptamer binding, further reducing potential sources of non-specific binding and detection (Fig. 1E). Combining a DNA aptamer for target biorecognition and the EG–gFETs for transduction forms the base of the graphene aptasensor array for ultrasensitive dopamine detection.

To confirm graphene channel biofunctionalization, the shift in the transistors transfer curves, i.e., the transconductance modulation expressed in source-drain current (I_DS_) changes for a particular gate voltage (V_GS_), was assessed after each functionalization step (Fig. 1F, G). Shifts from the baseline transfer curve measurements indicate charge carriers’ redistribution in the graphene channel due to electrostatic potential changes from surface modification. Measuring the gMTAs EG–gFETs transconductance in phosphate-buffered saline (PBS) produced ambipolar "V"-shaped transfer curves, typically reported for graphene FETs [21, 26, 48] (Fig. 1G). This transfer curve, symmetric about the charge neutrality point or Dirac point (V_DIRAC_), with a left branch (V_GS_ < V_DIRAC_) representing the excursion of the electrochemical potential (µ) in the valence band (p-branch) and a right branch (V_GS_ > V_DIRAC_) representing the excursion of µ in the conduction band (n-branch), is very distinct from semiconductor-based FETs and demonstrates the bipolar character of graphene transistors [16]. I_DS_ is at its minimum at the Dirac point and tracking the V_DIRAC_ value can be used as a proxy for transfer curve shifts (Fig. 1G). In the as-fabricated EG–gFETs, V_DIRAC_ was observed at positive V_GS_ (Fig. 1G), a consequence of unintentional p-doping during the cleanroom lithographic processes. The first measurement with the bare graphene channel in PBS, i.e., before any biofunctionalization step, showed V_DIRAC_ between 0.5 and 0.6 V in all transistors. Gate electrode passivation with DDT produced a sharp negative shift of V_DIRAC_ of − 246 ± 51 mV (Fig. 1C). This shift is attributable to the formation of a SAM covering the gold electrode, creating an excess of positive charges in the solution from the dipole moment reorientation of the alkanethiols [28, 49]. The addition of the crosslinker PBASE produced a slight positive shift of V_DIRAC_ of 23 ± 23 mV (Fig. 1G) as PBASE leads to electron withdrawal (p-doping) [28, 45]. However, adding the DNA aptamer induced a sharp positive shift of V_DIRAC_ of 113 ± 31 mV (Fig. 1G). This shift is explained by increased negative charges close to the graphene channel due to DNA’s negatively charged phosphate backbone. The final step in the functionalization process, blocking free PBASE with ETA, produces a slight negative shift of V_DIRAC_ of − 12 ± 11 mV (Fig. 1G). The final value of V_DIRAC_ for each functionalized EG–gFET in the gMTA, was then used as the baseline for dopamine detection experiments with the gMTA aptasensor.

Although transfer curve measurements help track and compare the success of the biofunctionalization process across different gMTAs, the aptamer immobilization process was further assessed with X-ray photoelectron spectroscopy (XPS) by comparing experimental peak parameters for O 1 s, C 1 s, N 1 s, P 2p from graphene samples incubated with either PBASE or PBASE followed by the aptamer (Fig. 1H, Table 1). The high-intensity C 1 s peak in the PBASE sample comes from the graphene present on the substrate, whereas the O 1 s and N 1 s peaks come from PBASE’s ester group. PBASE also contributes with carbon but only marginally when compared with graphene. The small atomic percentage of P 2p in the PBASE sample is attributed to the silicon wafer. In the PBASE + aptamer sample, there is a visible increase in O 1 s, C 1 s, and P 2p atomic percentages relative to the PBASE sample. The O 1 s and C 1 s increases are attributed to the nucleobases and the sugar units that form nucleotides in DNA. The significant P 2p atomic percentage increase is also a distinctive DNA signature since each nucleotide has a phosphate group that forms the phosphate backbone in DNA. Further analysis of the peaks of interest and the graphene-PBASE-aptamer bond structure can be found in Additional file 1: Fig. S3.

**Table 1:** XPS peak parameters for O 1 s, C 1 s, N 1 s, and P 2p for PBASE and PBASE + aptamer on graphene substrates

	Height CPS	FWHM eV	Area (P) CPS.eV	Atomic %
PBASE				
O 1* s*	138,359.14	3.08	470,188.90	25.77
C 1* s*	137,259.15	2.92	509,376.12	70.79
N 1* s*	7954.53	3.30	35,815.32	3.14
P 2*p*	489.15	2.50	3105.26	0.30
PBASE + Aptamer				
O 1* s*	155,742.38	3.02	541,535.84	31.25
C 1* s*	155,495.53	2.97	557,787.48	73.41
N 1* s*	5938.77	4.53	36,951.10	3.07
P 2*p*	1655.57	8.32	12,324.35	1.25

### Dopamine detection in physiological buffers

**Fig. 2:**
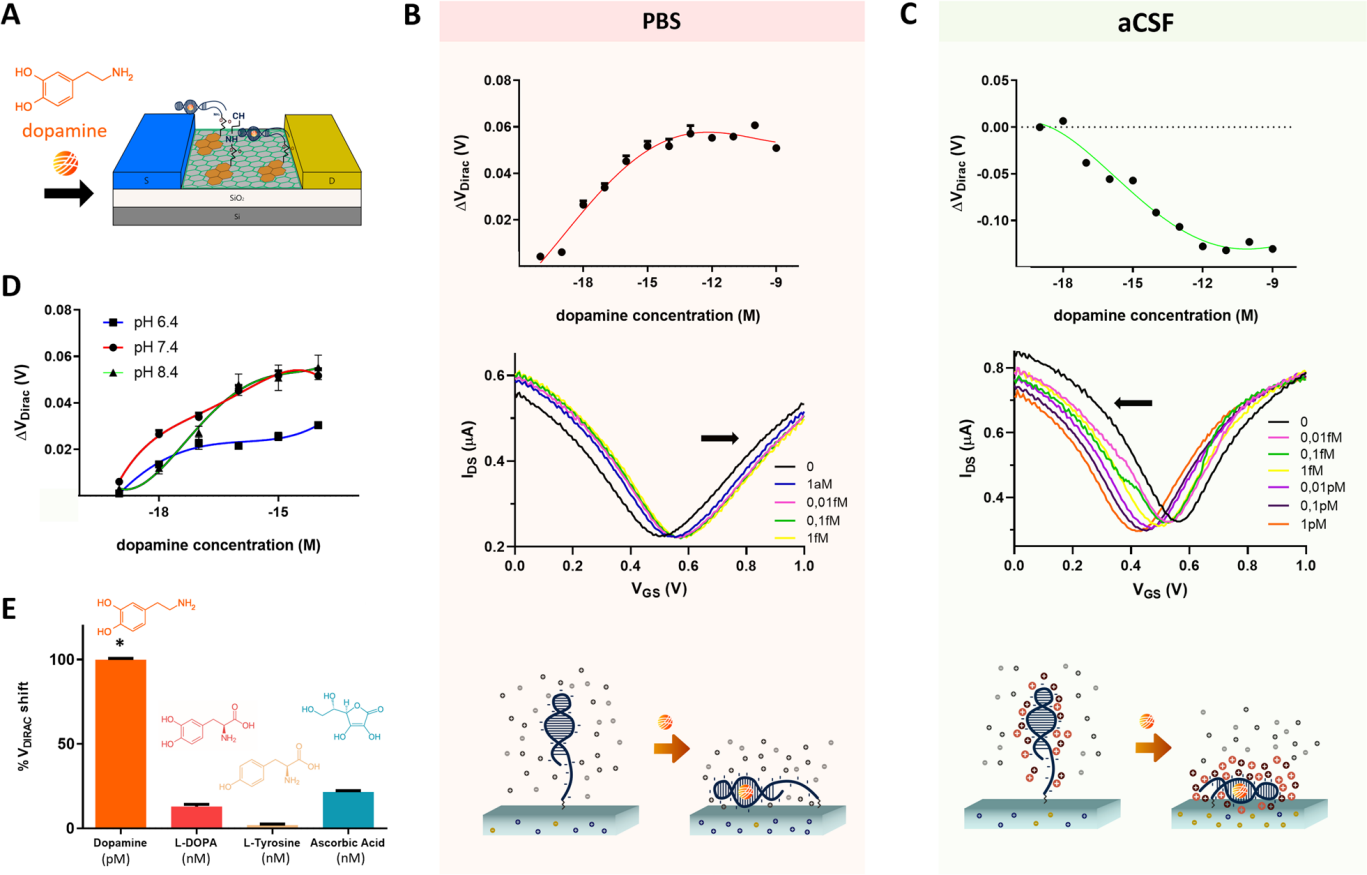
Dopamine detection in physiological buffers with gMTAs. **A** Schematic illustration of aptamer structure reorientation close to an EG–gFET graphene channel upon dopamine binding. **B** Calibration curve for dopamine detection in 1 × PBS (data is mean ± sem, with 4th order polynomial line fit) (top). Representative transfer curve shifts as a function of increasing dopamine concentrations for the linear detection range of one EG–gFET in 1 × PBS. V_DIRAC_ moves towards positive gate voltages (V_GS_) (black arrow indicates the direction of V_DIRAC_ shifts) (middle). Illustration of the hypothesized reorientation of the aptamer's backbone negative charges close to the gFETs upon dopamine binding in 1 × PBS, leading to electrostatic repulsion in the graphene channel (bottom). **C** Calibration curve for dopamine detection in 1 × aCSF (data is mean ± sem, with 4th order polynomial line fit) (top). Representative transfer curve shifts as a function of increasing dopamine concentrations for the linear detection range of one EG–gFET in 1 × aCSF, with V_DIRAC_ moving towards negative gate voltages (V_GS_) (black arrow indicates the direction of V_DIRAC_ shifts) (middle). Illustration of the hypothesized reorientation of the aptamer's structure in aCSF upon dopamine binding, with the increased attraction of positive charges closer to the graphene channel, leading to negative shifts of V_DIRAC_ (bottom). **D** Calibration curves for dopamine detection in 1 × PBS at pH 6.4 (blue), 7.4 (red) and 8.4 (dark green) (data is mean ± sem, with 4th order polynomial line fit). **E** Comparative responses of gMTAs to 1 pM dopamine, 1 nM L-DOPA, 1 nM L-tyrosine and 1 nM ascorbic acid in 1 × PBS (normalized data is mean + sem; One-way ANOVA, *p < 0.0001)

For initial validation of the gMTAs dopamine detection in ultra-low concentrations, as well as to establish calibration curves, in vitro experiments were performed in undiluted phosphate-buffered saline (1 × PBS) and artificial cerebrospinal fluid (1 × aCSF) (Fig. 2A). Dopamine prepared from stock solution was added to these buffers in increasing concentrations from zM (10^–20^) to nM (10^–9^). The selectivity of the gMTA aptasensor against dopamine synthesis molecules and biological interferents was also assessed.

#### Dopamine detection in PBS

A calibration curve for the gMTAs response (ΔV_DIRAC_) to dopamine was obtained by serially incubating solutions of increasing dopamine concentrations in undiluted phosphate-buffered saline (1 × PBS) (Fig. 2B). V_DIRAC_ measurements were offset-corrected by 19.9 mV, which was the mean ΔV_DIRAC_ obtained for blank samples, i.e., samples prepared from dilution of dopamine stock solution but not containing dopamine molecules (Additional file 1: Fig. S4). Adding dopamine to the gMTAs led to a significant V_DIRAC_ shift of approximately 26 ± 1 mV for a concentration as low as 1 aM (10^–18^) (Fig. 2B), a record limit-of-detection (LOD) for dopamine. The intrinsic variance of the gMTAs’ measurements was also calculated by assessing their response to blank samples over time (Additional file 1: Fig. S5). The obtained coefficient of variation (CV) for ΔV_DIRAC_ was 1.13%, which is indicative of the gMTA’s high stability in the presence of solution ions and well below the observed 26 ± 1 mV V_DIRAC_ shift obtained for 1 aM (10^–18^) dopamine concentration. From 1 aM (10^–18^) dopamine concentration, gMTAs presented a linear detection range up to 1 fM (10^–15^), with linear increases of V_DIRAC_ as a function of dopamine concentration with a 9.5 mV/decade sensitivity (Fig. 2B). To establish the calibration curve for dopamine detection in PBS, the incubation time for each sample was 1 h. However, similar responses can also be obtained with just 5 min of sample incubation (Additional file 1: Fig. S5). The positive shifts in V_DIRAC_ with increasing concentration of dopamine are hypothesized to occur due to reorientation of the negative charges of the DNA aptamer phosphate backbone near the EG–gFETs’ channels upon dopamine binding [50] (Fig. 2B). These charges, acting as counter-ions, would raise the energy of the graphene electron bands, effectively shifting the electrochemical potential, µ, down in the valence band. Consequently, the charge neutrality point (V_Dirac_) is found at more positive gate voltages, required to bring µ back to the Dirac point.

The dopamine LOD of 1 aM (10^–18^) obtained with our gMTAs is 3 orders of magnitude lower than the lowest LOD ever reported for any dopamine sensor, currently at 0.5 fM (10^–15^) [12, 13], and several orders of magnitude lower than the LOD attained with the majority of previous methodologies, as summarized in Table 2. Additionally, the sensors currently presenting the lowest dopamine LOD, although based on organic field-effect transistors [12] or graphene-coated electrodes [13], rely on voltammetric electrochemical measurements or require the addition of labels/reporters. The gMTA aptasensor is a label-free detection device requiring low operational voltage with high transconductance due to the high gate capacitance from the electrical double layers (EDLs) at the graphene-electrolyte and electrolyte-gate interfaces. The sensitivity of our sensor results from a combination of factors: (i) the EG–gFETs high 2D conductance, deriving from CVD graphene’s single-layer high electronic mobility and relatively high carrier density [18, 20]; (ii) the cleanroom fabrication process carefully developed to preserve graphene’s electronic properties, while simultaneously passivating all other device areas [44]; (iii) the graphene transistor channel direct exposure to the liquid medium containing the target and the EDLs formation [21, 51]; (iv) the aptamer’s affinity to dopamine and its ability to operate within the Debye length [46, 47, 52].

**Table 2:** Comparison of different biosensors for dopamine detection

Biosensor configuration	Detection method	LOD	Dynamic Range	Refs.
gFET + aptamer	Transconductance	1 aM	1 aM–100 µM	This work
Glassy carbon electrode (GCE)/rGO-polyurethane	DPV	10 pM	0. 1–1.15 nM	[53]
conducting Polymer + rGO + aptamer	Voltammetry	78 fM	1 pM–160 nM	[54]
Palladium NPs-loaded Carbon nanofibers	DPV	0.2 µM	0.5–160 µM	[55]
Conducting polymer nanotubes liquid gated-FET + aptamer	Transconductance	100 pM	–	[56]
In_2_O_3_ FET + aptamer	Transconductance	1 fM	1 fM–10 pM	[47]
Gold electrode + aptamer	Amperometry	62 nM	0.1–1 μM	[57]
Fluorescence aptasensor	Voltammetry/fluorometric	80 pM	100 pM–10 nM	[58]
MoS_2_ QDs MoS_2_ Nanosheets + aptamer	FRET	45 pM	0.1–1000 nM	[59]
Organic electrochemical transistor (OECT) + split aptamer	Amperometry	0.5 fM	5 fM–10 pM	[12]
FRET quenching biosensor + aptamer	FRET	0.12 μM	0–15 μM	[46]
Carbon-dot–tyrosinase bioprobe	Fluorescence	60 nM	0.1–6.0 μM	[60]
Microfluidic plasma separator	Plasmonics	100 fM	–	[61]
Tryptophan-modified electrodes	FSCV	2.48 nM	–	[62]
Au-coated arrays of micro pyramid structures (Au-MPy)	CV	0.50 nM	0.01–500 µM	[63]
Boron-doped diamond electrode (CB-Nafion/p-BDD)	CV	54 nM	0.1–100 μM	[64]
Single-Atom Ruthenium Biomimetic Enzyme (Ru-Ala-C3N4)	Catalytic/amperometry	20 nM	0.06–490 μM	[65]
Chitosan/graphene quantum dots thin film	SPR	1.0 fM	0.1 fM–1 pM	[66]
Fe_3_O_4_-AuNPs coated FET	Transconductance	3.3 nM	1–120 µM	[67]

*CV* Cyclic Voltammetry, *DPV* Differential Pulse Voltammetry, *FET* Field-effect transistor, *FRET* Fluorescence resonance energy transfer, *FSCV* Fast Scanning Cyclic Voltammetry, *LSPR* Localized Surface Plasmon Resonance, *NPs* Nanoparticles; *QDs* Quantum dots; *rGO* reduced graphene oxide; *SPR* Surface Plasmon Resonance

#### Dopamine detection in aCSF

To further validate the ultrasensitivity of our gMTAs in vitro, calibration curves for dopamine detection in undiluted artificial cerebrospinal fluid (1 × aCSF) were acquired (Fig. 2C). aCSF does not contain other molecules found in biological CSF – such as amino acids, proteins, and hormones – but it closely matches biological CSF’s electrolytic profile and osmolarity. The observed LOD in 1 × aCSF was 10 aM (10^–17^) (Fig. 2C), which is one order of magnitude higher than that observed in 1 × PBS. This difference was expected because the Debye length is lower in aCSF compared with PBS due to a higher ionic strength at the same pH, attributable to additional divalent cations in the solution. Nevertheless, while most reported dopamine sensors were not tested in aCSF, the observed LOD is still 3 orders of magnitude lower than the lowest previously reported LOD for a dopamine sensor in aCSF at 10 fM (10^–14^) [47]. The dynamic detection range is also broader in aCSF compared with PBS, going up to 100 nM (10^–11^) with a 22 mV/decade sensitivity (Fig. 2C).

Contrary to the calibration curve in 1 × PBS, adding dopamine to the gMTAs in 1 × aCSF led to negative shifts of V_DIRAC_, which means that the graphene electrochemical potential moved up in energy relative to the density of states (n-doping). The sign of V_DIRAC_ shifts depends on several mechanisms involving interactions between the probe and the target and between the electrolytic solution and the probe and target [20]. Thus, one possible explanation for the observed negative shift is that the secondary structure of the dopamine aptamer is different in aCSF compared with that in PBS, bringing positively charged molecular regions towards the graphene channel upon dopamine binding (Fig. 2C). These results lend further support to previous observations reporting potential alterations of the secondary structure of this aptamer in the presence of Ca^2+^ and Mg^2+^, which are present in aCSF [46, 50]. The excess of positive charges near the graphene channel upon dopamine binding may also explain the significant increase of the sensitivity per decade in aCSF, because each detection event may add multiple positive charges within the Debye length.

Although aptamers typically present high stability at variable pH, changes in the ionic composition of the electrolytic solution can alter aptamers’ conformation and FETs performance [68, 69], as also suggested from the aCSF data. Thus, dopamine detection was assessed in 1 × PBS solutions with a pH of either 6.4 or 8.4 (Fig. 2D). The 1 aM LOD was still observed at pH 6.4 and 8.4, but with lower magnitude responses for this dopamine concentration when compared with detection at physiological pH (7.4). Detection at pH 8.4 presented a similar 3 concentration decades dynamic range (10.5 mV/decade sensitivity) to detection at pH 7.4 (9.5 mV/decade sensitivity), while detection at pH 6.4 displayed a significantly lower sensitivity (5.5 mV/decade). These results confirm that the electrolytic solution’s ionic composition can interact with the biorecognition probe or the EDLs and change the transistors’ response.

*Selectivity assessment*. Evaluating the selectivity of novel biosensors is paramount for successful real-world applications with biological samples where the target molecule is mixed with various other molecules. Brain dopamine or dopamine in brain-derived samples, such as biological CSF, occurs at minute concentrations and is mixed with other neurotransmitters, amino acids, and proteins. Thus, to test the selectivity of the gMTAs, their response to dopamine was compared with the response to molecules in the dopamine synthesis pathway, including L-Dopa and L-Tyrosine, chemically similar to dopamine, and ascorbic acid, a ubiquitous biological interferent typically occurring in higher concentrations than neurotransmitters. The latter is involved in norepinephrine synthesis from dopamine and is likely to co-occur with dopamine in the brain [70]. The response of the gMTAs to the other tested molecules, even when present in high concentrations (1 nM), was negligible compared with the response to low concentrations of dopamine (1 pM) (Fig. 2E), confirming the aptamer’s specificity and high affinity to dopamine. The response to other monoamine neurotransmitters, such as serotonin and norepinephrine, was not tested, but a previous report has demonstrated that this aptamer has reduced affinity for those neurotransmitters [47].

### Dopamine detection in an animal model of Parkinson’s disease

Biosensors developed and optimized based on in vitro assays tend to underperform in biological samples, losing sensitivity and selectivity. This underperformance, also observed in previous ion-sensitive dopamine biosensors [12, 13], is usually due to a high number of interferent molecules and ions typically not found in optimized buffers, the reduction of the Debye length due to the increased shielding effect of the biological solution’s additional counter-ions [71], and sensor’s intrinsic variability masking small responses. Thus, to evaluate the performance of our gMTAs and our multi-sampling approach to overcome measurement variability in a physiological scenario, dopamine detection experiments were performed in biological CSF and brain homogenate samples.

A reserpine-induced mouse model of Parkinson’s Disease (PD) was used for these experiments. This animal model has been instrumental in elucidating the role of abnormal dopamine levels in PD symptomatology and used to develop critical therapeutics for PD that modulate these levels, such as L-DOPA administration [72, 73]. Reserpine is an irreversible and non-selective inhibitor of the vesicular monoamine transporters VMAT1 and VMAT2, and its systemic administration impairs monoamine uptake and storage in neuronal cells leading to the rapid depletion of brain dopamine from neuronal synapses [74–76]. This depletion leads to impaired motor function resulting in behavioral symptoms similar to PD caused by the loss of dopaminergic neurons in the human brain [75, 76]. As expected, 8 h post-administration, all our reserpine-treated mice displayed severe akinesia, postural instability, and tremors, while controls (vehicle-treated mice) displayed standard motor and exploratory behavior.

#### Dopamine detection in biological CSF

**Fig. 3:**
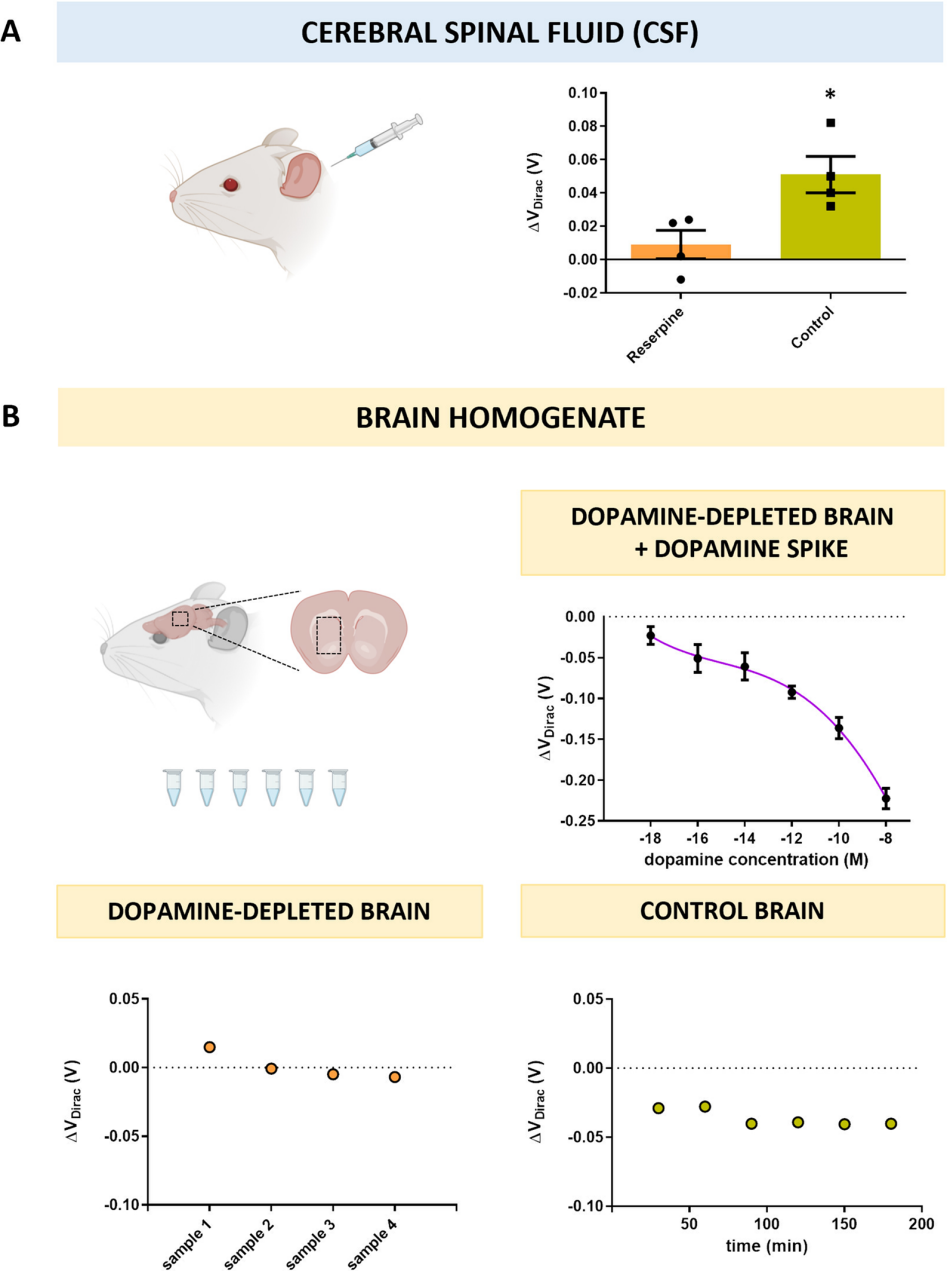
Dopamine detection with gMTAs in biological samples from a mouse model of Parkinson's Disease (PD). **A** Response of gMTAs to biological cerebral spinal fluid (CSF) samples obtained from PD (reserpine) and control mice (data is mean ± sem; paired *t*-test, *p < 0.05). **B** gMTAs response to dopamine-depleted brain homogenate samples obtained from PD mice spiked with increasing dopamine concentrations (top) (data is mean ± sem, with 4th order polynomial line fit). gMTAs response to sequentially incubated dopamine-depleted brain homogenate samples obtained from PD mice (bottom-left). gMTAs response to brain homogenate sample obtained from control mice incubated continuously for 3 h

To test if our gMTAs could detect small changes in dopamine concentration from small volume biological samples, their ability to differentiate CSF samples obtained from control (vehicle-treated) and parkinsonian (reserpine-treated) mice was assessed (Fig. 3A). Dopamine levels in CSF can serve as a biomarker for PD since their significant decrease in patients has been previously observed [77, 78]. CSF was extracted by *cisterna magna* puncture [79], a method similar to subarachnoid puncture used in humans for clinical diagnosis [80]. Pairs of 2µL CSF samples obtained from reserpine-treated (dopamine-depleted) and control animals were incubated sequentially, in this order, on each gMTA. A five-fold response difference was observed when comparing V_DIRAC_ shifts of samples from parkinsonian animals (reserpine, mean ΔV_DIRAC_: 9 ± 8 mV) with those of control animals (mean ΔV_DIRAC_: 51 ± 11 mV) (Fig. 3A). Of note, CSF samples were directly incubated in the gMTAs after extraction without the need for any sample preparation, and measurements were acquired after 10 min of incubation only. This result is of great importance for developing fast-acting point-of-care devices.

Additionally, the gMTAs displayed significantly higher sensitivity than previously developed biosensors for dopamine detection in CSF while requiring significantly lower sample working volumes [81–83]. The ability of our gMTAs to detect minimal changes in dopamine concentration in small volume biological samples is essential because limiting the sample volume is a desirable feature for real-world applications that can measure dopamine from scarcely abundant samples such as CSF. Since PD is a progressive disorder and dopamine loss starts before motor symptoms arise, developing a sensor that can detect small changes in dopamine from these samples is highly relevant for developing earlier and improved diagnostics.

#### Dopamine detection in brain homogenate

Finally, to test our gMTAs’ dopamine detection in a media that more closely mimics the complexity of the brain’s extracellular space, homogenate brain samples from reserpine-treated (dopamine-depleted) animals were collected and spiked with increasing dopamine concentrations. A series of samples were prepared from stock solution, from aM (10^–18^) to nM (10^–8^) (Fig. 3B). The gMTAs still displayed high sensitivity for dopamine detection, overcoming sensitivity losses typically observed in such complex media (Fig. 3B). The observed LOD was 1 aM (10^–18^), and a wide dynamic range from 1 aM (10^–18^) to 100 µM (10^–8^) was obtained (Fig. 3B).

Two additional control experiments were performed to confirm the selective detection of dopamine in brain homogenates by the gMTAs. First, samples from reserpine-treated animals without dopamine spiking were incubated in the gMTAs. This experiment would confirm that the previously observed changes in transconductance were due to the selective detection of dopamine and not of other molecules present in the brain homogenates (Fig. 3B). As expected, no significant V_DIRAC_ shifts away from baseline values were observed, denoting the absence of dopamine detection (Fig. 3B). Then, one homogenate sample pooled from 2 control animals’ brains was continuously incubated in one gMTA for 180 min, and measurements were taken every 30 min. This experiment was performed to confirm that the observed significant shifts in V_DIRAC_ from the dopamine spiked samples were due to the presence of dopamine and not a time-dependent variation in transconductance induced by other causes. A sharp 29 mV V_DIRAC_ shift was observed for the first measurement, after 30 min incubation, attributed to the presence of dopamine in the control brains (Fig. 3B). Then, no significant V_DIRAC_ shifts between measurements were observed for the remaining 150 min (Fig. 3B). The fact that dopamine can be reliably detected in ultra-low concentrations in samples resembling the brain’s extracellular space with the gMTAs can also extend the applications of our biosensor to future in vivo measurements requiring detection of localized dopamine release in complex biological scenarios.

## Conclusions

The graphene aptasensor multitransistor array (gMTA) for dopamine detection proposed here, combining an array of graphene field-effect transistors with a selective DNA aptamer, achieved ultrasensitive and stable dopamine detection in various media from physiological ionic-strength buffers to complex biological samples. Notably, the gMTAs detected dopamine changes in small working volume CSF samples from a Parkinson’s Disease (PD) animal model, which can pave the way for novel point-of-care diagnostic devices to detect abnormal levels of dopamine in PD, and in other dopamine-dependent brain disorders such as Alzheimer’s Disease, and substance addiction disorders. Using numerous EG–gFET aptasensors in an array configuration further allows future multiplexed measurements of different biomarkers in one single gMTA through the localized biofunctionalization of individual EG–gFETs. Lastly, the fabrication at the wafer level lends itself to developing different array configurations, including with higher EG–gFETs count, which may be relevant for a high-throughput assessment of localized dopamine release. Increased sensors’ spatial density and tracking real-time independent localized measurements with single-cell resolution from each EG–gFET or subsets of transistors in the arrays are future features that can be realized without significant changes to the overall fabrication process. This possibility is especially relevant for fundamental neuroscience or pharmaceutical studies of brain disorders requiring measurements from ex vivo brain slices from animal models or human patients’ IPSC-derived cell cultures and in vivo monitoring in intact brains. As the proposed sensor is further tested in other biologically relevant samples and in in vivo scenarios, it can ultimately help elucidate our understanding of the brain and promote the development of improved diagnostics and therapeutics for brain disorders.

## Materials and methods

### Materials and reagents

3,4-dihydroxyphenethylamine hydrochloride (Dopamine hydrochloride), 3,4-Dihydroxyl-L-phenylalaline (L-DOPA) (98% TLC), L-Tyrosine (98% HPLC), L-Ascorbic acid (99%), Phosphate-buffered saline (PBS) tablets, N,N-Dimethylformamide (DMF) (99.9% HPLC), 1-Pyrenebutynic acid N-hydroxy-succinimide ester (PBASE) (95%), 1-Dodecanethiol (DDT) (98%), Ethanolamine (ETA) (98%), Sodium Chloride (NaCl), Potassium Chloride (KCl), Monosodium Phosphate (NaH2PO4), Sodium Bicarbonate (NaHCO3), Glucose, Calcium Chloride Dihydrate (CaCl2.2H2O), Acetic Acid Glacial, Hydrochloric acid (HCl) (35%), Sodium Hydroxide (NaOH) tablets, and Poly(methyl(meth)acrylate) (PMMA) (15 k M.W.) were purchased from Sigma-Aldrich. Acetone (99.5%), Ethanol (99.8%), and 2-Propanol (99.8% GC) were purchased from Honeywell. Magnesium Sulfate Heptahydrate (MgSO4.7H2O) was purchased from Merck. Photoresist AZ1505 (AZ) was purchased from MicroChemicals GmbH. RTV silicone elastomer (3140, Dowsil) and superglue (Loctite) were acquired from Farnel. DNA aptamer (5′-CGACGCCAGTTTGAAGGTTCGTTCGCAGGTGTGGAGTGACGTCG-3′) with a 5′ C6-amino link modification was synthesized by Stab Vida. Reserpine was acquired from Biogen. MilliQ water used in all experiments had a resistivity higher than 18 MΩ cm at 25 °C.

### Graphene multitransistor array fabrication

Graphene multitransistor arrays (gMTAs) were fabricated by building on our previously published processes for wafer-scale fabrication of electrolyte-gated graphene field-effect transistors (EG–gFETs) with an integrated receded gate electrode and improved multilayer dielectric passivation for biosensing applications [38, 44]. Briefly, single-layer graphene (SLG) was grown by thermal chemical vapor deposition (CVD) on 25 µm thick high-purity (99.999% purity) copper (Cu) foils in a three-zone quartz tube furnace (EasyTube ET3000, CVD Corp.). The 10 × 10 mm^2^ Cu substrates were initially treated with a mixture of FeCl_3_, HCl, and deionized water (DI) for 1 min in ultrasound and then placed in the furnace. The system was evacuated to approximately 2 mTorr and then filled with 250-sccm Argon (Ar, 99.999% purity) and 60-sccm Hydrogen (H_2_, 99.999% purity) gas mixture. Once the growth temperature and pressure were reached, methane (0.5 sccm), the carbon precursor, was introduced into the chamber. The growth was carried out at 1040 °C at 6 Torr for 25 min. After growth, SLG was protected by poly(methyl(meth)acrylate) (PMMA), and plasma ashing was performed to remove the graphene from the backside of the substrate. Raman spectroscopy was used to assess the quality of grown graphene (Additional file 1: Fig. S1).

For wafer-level fabrication of EG–gFETs, a 200 mm silicon (Si) wafer with 100 nm of thermal oxide was used as substrate. The wafer was sputter-coated with Chromium (Cr) (3 nm) as an adhesion layer, Gold (Au) (35 nm) as the conductive layer, and an alumina (Al_2_O_3_) (20 nm) capping. The source, drain, and gate electrodes were patterned by optical lithography and etched by ion milling. A sacrificial layer (TiW, 5 nm; AlSiCu, 100 nm; TiWN, 15 nm) was sputtered and patterned via lift-off, exposing the channel region and the source and drain electrodes for graphene transfer. Previously grown SLG, as described above, was transferred onto the wafer, patterned with optical lithography, and dry-etched with oxygen plasma. The sacrificial layer was then removed by wet etch. A protective layer (Ni, 10 nm; AlSiCu 30 nm; TiWN 10 nm) was sputtered and patterned by sonication-free lift-off to work as a stopping layer for reactive ion etching (RIE) at the graphene channel and gate electrode. A 250 nm multi-stack passivation layer of SiO_2_ and Si_3_N_4_ was grown by plasma-enhanced CVD and patterned by RIE. Finally, the stopping layer was dry-etched to expose the graphene channel and gate electrode. The EG–gFETs were characterized electrically at the wafer level with an automated probe station by measuring the resistance between each transistor’s source and drain electrodes at a fixed voltage of 1 mV or a fixed current of 1 μA. gMTAs with transistors with resistance above 2.5 MΩ were discarded from further experiments. The wafer was finally coated with photoresist for protection and diced into individual 4.5 × 4.5 mm gMTA chips. The chips were then glued onto custom-designed printed circuit boards (PCB) for electronic interfacing, and their interconnection pads were wire-bonded with gold wires to the PCB pads. Finally, the wires and connection pads were protected with a silicone elastomer.

### Graphene transistors functionalization

EG–gFETs in each gMTA chip were first incubated for 4 h in 2 mM 1-Dodecanethiol (DDT, 2 mM in ethanol) for gate passivation, then cleaned with DI water and dried under N_2_ flow. Then, 20 µL of the pyrene-derived crosslinker 1-Pyrenebutyric acid N-hydroxysuccinimide (PBASE, 10 mM in dimethylformamide, DMF), were added to the gMTAs and incubated for 2 h in a humid chamber. A 44nt-long DNA aptamer (5′-CGACGCCAGTTTGAAGGTTCGTTCGCAGGTGTGGAGTGACGTCG-3′), previously selected for high affinity to dopamine [47], with a 5′ amino-link termination, was initially diluted in MilliQ water to 20 µM, heated to 95 °C for 5 min, and then allowed to cool to room temperature. Then, 20 µL of aptamer solution was added to the gMTAs and incubated for 16 h in a humid chamber in the dark. 20 μL of ethanolamine (ETA, 100 mM in DI water) were incubated in the gMTAs for 30 min to bind to PBASE via the amine termination and block any remaining PBASE that did not bind to the aptamers. Finally, gMTAs were rinsed with DI water and dried under N_2_ flow. The transconductance of each EG–gFET aptasensor in the arrays was measured at each step of the functionalization process by applying a source-drain voltage (V_DS_) of 1 mV and measuring source-drain current (I_DS_) in gate-source voltage (V_GS_) sweeps between 0 and 1 V.

### X-ray photoelectron spectroscopy (XPS)

X-ray photoelectron spectroscopy (XPS) analysis was performed with an ESCALAB 250Xi system (Thermo Scientific). A thermal p-doped silicon oxide wafer was coated with a photoresist and cut in 1 mm^2^ substrates. Previously grown SLG was transferred to the substrate, after which samples were incubated with the PBASE crosslinker (for 2 h). Half of the samples were then incubated with the dopamine-specific aptamer (for 16 h). The PBASE and aptamer incubation times were the same as for the transistor’s graphene functionalization. Survey scans were performed from 0 to 1350 eV with a pass energy of 50 eV. High-resolution spectra for carbon, nitrogen, oxygen, and phosphorous were completed for graphene + PBASE and graphene + PBASE + aptamer samples. Advantage software (Thermofisher) was used for peak fitting and calculating atomic percentages.

### Dopamine detection in physiological buffers

Dopamine hydrochloride (3,4-dihydroxyphenethylamine hydrochloride) in powder form was first prepared into a stock solution of 10 mM in either phosphate-buffered saline (PBS) (mM: 137 NaCl, 2.7 KCl, 10 KH_2_PO_4_, 1.8 NaH_2_PO_4_) or artificial cerebral spinal fluid (aCSF) (mM: 119 NaCl, 2.5 KCl, 1.2 NaH_2_PO_4_, 24 NaHCO_3_, 12.5 glucose, 2 MgSO_4_.7H_2_O and 2 CaCl_2_.2H_2_O, 300–310 mOsm/L) at pH 7.2–7.4. Solutions of different dopamine concentrations in 1 × PBS or 1 × aCSF were prepared by diluting the stock solutions from zM (10^–20^) to nM (10^–9^). Baseline transconductance for EG–gFETs in each gMTA aptasensor was measured in PBS or aCSF not containing dopamine by applying a V_DS_ of 1 mV, and sweeping V_GS_ between 0 and 1 V. Following baseline measurements, 20 μL samples of each dopamine concentration in either buffer were incubated in the gMTAs**.** Between samples, gMTAs were rinsed with DI water, and transconductance measurements were taken in 1 × PBS or 1 × aCSF accordingly, with the same voltage parameters used for baseline acquisition. More than 500 EG–gFETs from over 30 gMTAs provided measurements for the PBS and aCSF calibration curves, with each concentration for each electrolytic buffer being incubated in at least 4 gMTAs (minimum 80 EG–gFETs per concentration per buffer).

For the pH experiments, dopamine dilutions from 0.1 aM (10^–19^) to 10 fM (10^–14^), prepared as described above, were adjusted for a final pH of either 6.4 or 8.4 by adding droplets of 2 M HCl or 0.5 M NaOH respectively. Sample incubation and transconductance measurements were performed as described above.

L-DOPA (L-3,4-dihydroxyphenylalanine), L-Tyrosine, and ascorbic acid were diluted in 1 × PBS to 1 nM (10^–9^) concentration. Then, 20 μL samples of each solution were incubated in the gMTAs. After DI water rinse, transconductance measurements were taken in 1 × PBS with the same voltage parameters described above. Samples from each solution were incubated in at least 2 gMTAs (minimum 40 EG–gFETs per solution).

### Dopamine detection in biological samples from PD mouse model

Mice (n = 8) were injected with reserpine (5 mg/kg, i.p.) dissolved in 1% glacial acetic acid and diluted in 0.9% saline. Control mice (n = 6) were injected with vehicle solution not containing reserpine. 8 h post-administration, all mice were anesthetized with avertin (tribromoethanol, 20 mg/mL; 0.5 mg/g, i.p.), placed in a stereotaxic frame for head fixation, and CSF was collected by *cisterna magna* puncture. CSF samples were inspected for blood contamination, frozen in liquid nitrogen, and stored at – 80 °C. Mice were then perfused transcardially with 0.9% saline to remove circulating blood, and the brain was quickly removed. The striatum brain region on both hemispheres was dissected, frozen in liquid nitrogen, and stored at – 80 °C.

For dopamine detection in CSF, frozen samples were thawed on ice, and 2 µL samples were placed directly on the gMTAs. For every gMTA, transconductance measurements were first acquired for a CSF sample from a reserpine-treated animal incubated for 10 min. Following rinsing with PBS, a sample from a control animal was incubated in the same gMTA for 10 min. The EG–gFETs transconductance was measured by applying a V_DS_ of 1 mV and sweeping V_GS_ between 0 and 1 V.

Previously dissected striatum brain samples were first thawed on ice for dopamine detection in brain homogenate. Samples from two brains (a total of 4 hemispheres) were pooled in a single Eppendorf tube with 1 × aCSF (pH 7.4) added at 5 μL/mg for homogenization with a brain tissue homogenizer. Samples were then centrifuged at 14,000 rpm, for 15 min at 4 °C. Dopamine hydrochloride diluted in 1 × aCSF was added to 10 µL of homogenate supernatant samples from reserpine-treated animals on a 1:1 volume ratio for final dopamine concentrations ranging from 1 aM (10^–18^) to 10 nM (10^–8^). Each 20 µL sample was incubated for 20 min in one gMTA and then rinsed with 1 × PBS. Transconductance measurements were taken before rinsing with the same voltage parameters described above.

As negative controls, 20 µL brain homogenate samples pooled from two reserpine-treated animals diluted in aCSF on a 1:1 volume ratio without dopamine added were serially incubated in one gMTA for 20 min. Transconductance measurements and rinsing between samples were performed as described above. One 20 µL brain homogenate sample pooled from two control animals diluted in aCSF on a 1:1 volume ratio was incubated continuously for 3 h in one gMTA. Transconductance measurements were taken every 30 min without rinsing with the same voltage excursion as above.

### Statistical analysis

Statistical analysis was performed with either GraphPad Prism 9 for Windows (Graphpad Software) or Matlab ver. 2021a (Mathworks). Statistical details can be found in figure legends.

## Supplementary Information

**Additional file 1: Figure S1.** Raman spectrum of CVD grown single-layer graphene. **Figure S2.** Averaged response from measurement replicates of a single gMTA chip. **Figure S3.** XPS peak fitting for PBASE and PBASE + aptamer samples. **Figure S4.** Blank samples measurements in PBS. **Figure S5.** Stability of gMTA measurements. **Figure S6.** Dopamine attomolar detection with short incubation time.

## Data Availability

The datasets used and/or analyzed during the current study are available from the corresponding authors upon reasonable request.
