# Supplementary material for: Ultrasensitive dopamine detection with graphene aptasensor multitransistor arrays
Source: J Nanobiotechnology. 2022 Nov 24;20:495. doi: 10.1186/s12951-022-01695-0 (PMC9694542; doi:10.1186/s12951-022-01695-0)
Supplement: Supplementary file 1 — Additional file 1: Figure S1. Raman spectrum of CVD grown single-layer graphene. Figure S2. Averaged response from measurement replicates of a single gMTA chip. Figure S3. XPS peak fitting for PBASE and PBASE + aptamer samples. Figure S4. Blank samples measurements in PBS. Figure S5. Stability of gMTA measurements. Figure S6. Dopamine attomolar detection with short incubation time. [file 12951_2022_1695_MOESM1_ESM.pdf]

Figure S1: Raman spectrum of CVD grown single-layer graphene

Raman spectroscopy was performed under laser excitation (λ = 532 nm), and spectra were acquired from 10 × 10 μm^2 samples of CVD-grown graphene sheets transferred onto 200mm Si/SiO_2 substrates. Figure S1 shows a characteristic Raman spectrum of a graphene sample from a graphene sheet grown for transistors' fabrication. Two clear peaks were observed at approximately 1580 cm^{-1} and 2680 cm^{-1}, corresponding to the characteristic G and 2D bands, respectively (1,2). A small broad peak at approx. 1350 cm^{-1}, corresponding to the D band, was also observed. 2D-to-G intensity ratio was approx. 1.4. These results confirm the presence of crystalline single-layer graphene in our samples (1).

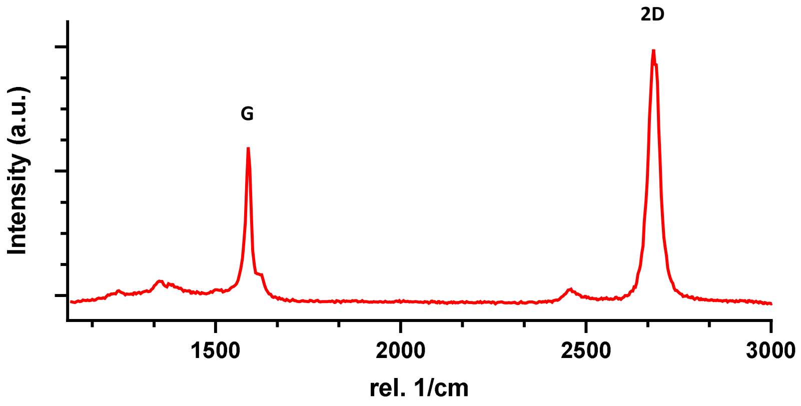

Figure S1. Raman spectrum of a CVD-grown graphene sample transferred onto a Si/SiO_2 substrate.

Figure S2: Averaged response from measurement replicates of a single gMTA chip

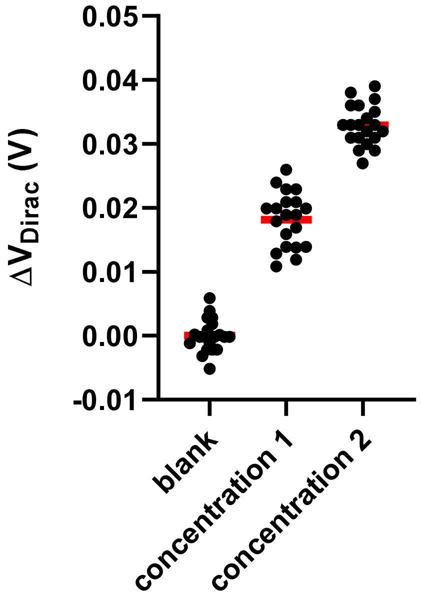

Figure S2. Individual transistor (black circles) and averaged (red lines) responses (ΔV_{Dirac}) from a single gMTA chip to a blank sample and to two samples with different concentrations of the analyte.

Figure S3: XPS peak fitting for PBASE and PBASE + aptamer samples

To confirm and further analyze the PBASE + aptamer bond for the implemented biofunctionalization process, high-resolution XPS spectra of C 1s, O 1s, and N 1s were fitted with Avantage data processing software (Thermo Fisher Scientific). Smart-type background subtraction was used for peak fitting, and quantification was done using sensitivity factors provided by the Avantage library. The XPS results support the successful binding of the DNA aptamer to PBASE in the substrate. Considering that the DNA aptamer binds to either the carbonyl group (C=O) or the carboxylate (C-O) part of the crosslinker (3), a reduction of these peaks, at approximately 287 eV and 285.5 eV, respectively, was observed in the PBASE + aptamer sample when compared with the PBASE sample (Fig. S2-A). The C-O bond peak from the PBASE sample became a C-N bond peak at approximately 286 eV in the PBASE + aptamer due to new contributions of C-O-C and C-OH bonds from the DNA aptamer sugar unit (Fig. S2-A) (4). A decrease of the N-(C=O)-O- bond peak at approximately 401.5 eV and an increase of the aromatic peak at approximately 399.4 eV was observed in the PBASE + aptamer sample when compared with the PBASE sample due to the amine termination of the DNA aptamer strand (Fig. S2-B). Two peaks were observed in the O 1s spectrum, a C=O bond peak at approximately 533 eV, which is from PBASE's aromatic ring, and an H-C-O bond peak at approximately 535 eV, which is from the PBASE's strand that links directly to the amino group from DNA (Fig. S2-C) (4). The H-C-O bond observed in the PBASE sample was not present in the PBASE + aptamer sample, likely due to the DNA aptamer amino group's binding to the crosslinker. For the same reason, the C=O bond peak in the PBASE + aptamer sample increases compared with the PBASE sample (Fig. S2-A).

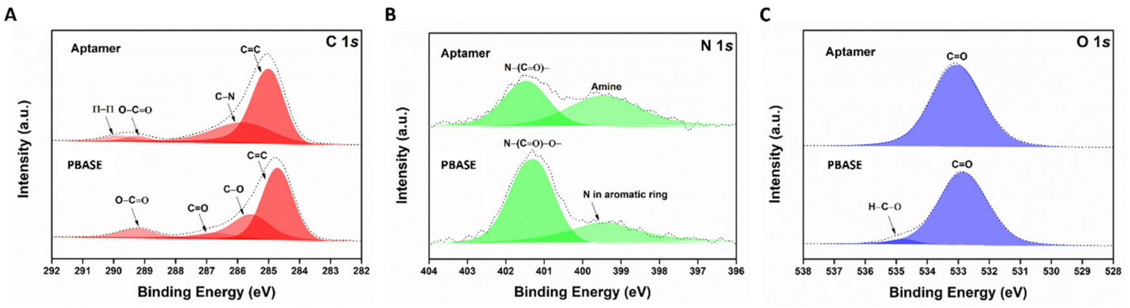

Figure S3. High-resolution XPS spectra from PBSAE and PBASE + DNA aptamer samples on graphene substrates for (A) Carbon (C 1s), (B) Nitrogen (N 1s), and (C) Oxygen (O 1s): acquired spectra (dashed lines) and peak fitting results (colored areas).

Figure S4: Blank samples measurements in PBS

To correctly determine the limit-of-blank of our gMTAs to offset the calibration curves, 20 μL samples not containing dopamine molecules but prepared from dopamine stock solution diluted to zM (10^{-21}) in 1 × PBS, were incubated for 20 minutes. Figure S3 shows the observed transconductance shifts from baseline (ΔV_{DIRAC}) for the blank samples, with an average of 19.9 ± 1.3 mV.

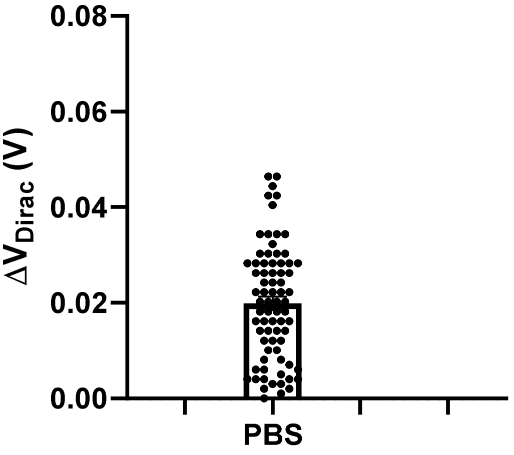

Figure S4. Transconductance shifts from baseline (ΔV_{DIRAC}) for blank samples not containing dopamine in 1 × PBS (left). Data is mean ± sem.

Figure S5: Stability of gMTA measurements

To assess the stability of our measurements, 20 µL 1X PBS samples were continuously incubated in one gMTA for 3 hours, and transconductance measurements were taken every 60 min. The coefficient of variation (CV) was calculated as the standard deviation to the measurements' mean ratio. A low CV of 1.13% was obtained, which indicates high stability.

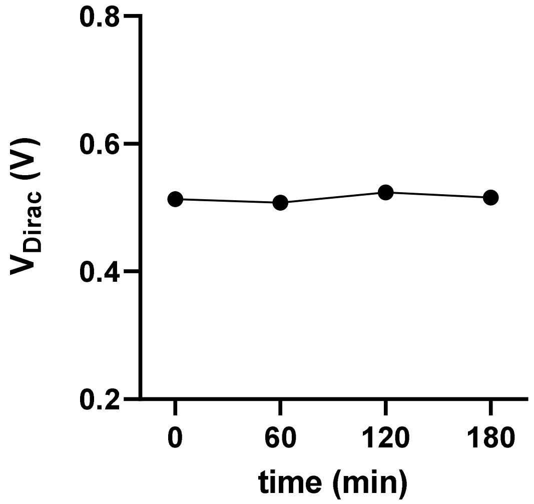

Figure S5. Transconductance (V_{DIRAC}) measurements of PBS samples over time. Data is mean ± sem.

Figure S6: Dopamine attomolar detection with short incubation time

To assess if the gMTAs could detect dopamine in ultra-low concentrations with short incubations times, dopamine samples in 1x PBS from 0.1 aM (10^{-19}) to 100 aM (10^{-16}) were incubated for 5 minutes and the responses compared with those obtained for 1 hour incubation. Figure S5 shows that transconductance measurements after 5 min sample incubation are comparable to those obtained for 1 hour sample incubation and that an LOD of 1 aM still present.

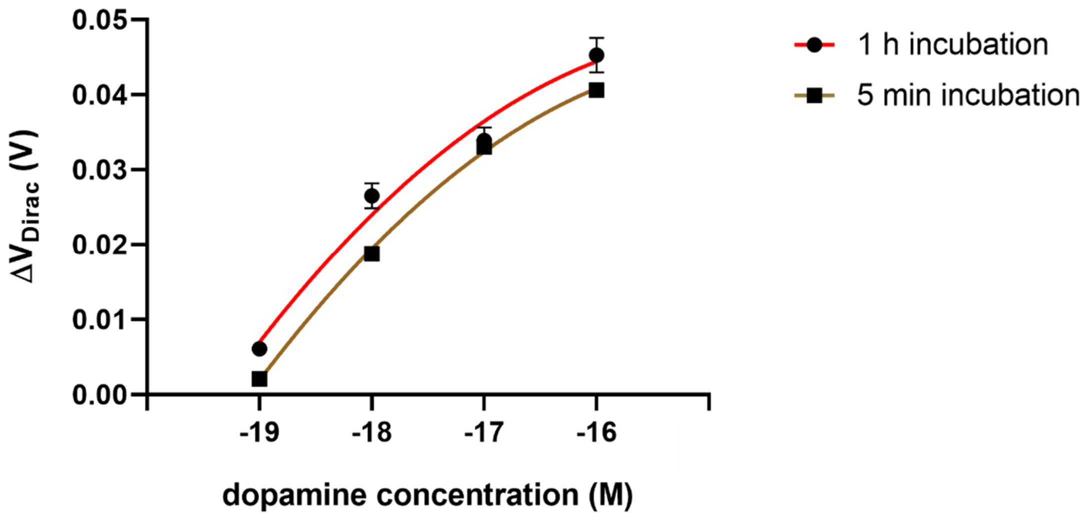

Figure S6. Calibration curves for dopamine detection in 1 × PBS with sample incubation times of 1h (red) and 5 min (brown) (data is mean ± sem, with 2nd order polynomial line fit).

References

1. Malard LM, Pimenta MA, Dresselhaus G, Dresselhaus MS. Raman spectroscopy in graphene. Physics Reports. 2009; 473(5-6): 51-87.

2. Childres I, Jauregui LA, Park W, Caoa H, Chena YP. Raman spectroscopy of graphene and related materials. In: New Developments in Photon and Materials Research. 2013. 403–18.

3. Fischer MJE. Amine coupling through EDC/NHS: a practical approach. Methods Mol Biol. 2010; 627:55-73.

4. Ptasińska S, Stypczyńska A, Nixon T, Mason NJ, Klyachko D V., Sanche L. X-ray induced damage in DNA monitored by X-ray photoelectron spectroscopy. J Chem Phys. 2008; 129(6): 065102.
